# Supplementary material for: Are carbon emissions trading and green financial instruments synergistic? -Comprehensive quantitative research based on content analysis
Source: PLoS One. 2024 Mar 7;19(3):e0298601. doi: 10.1371/journal.pone.0298601 (PMC10919601; doi:10.1371/journal.pone.0298601)
Supplement: S1 Data — (ZIP) [file pone.0298601.s001.zip › Data/地级市Z_F工作报告.zip!/╔╜╢1⁄2/╤╠╠¿╩╨/└┤╫╘╣1⁄2╓┌║┼í╛┬φ┐╦╩2╛▌═°í┐_2015.docx]

各位代表：

现在，我代表市人民政府向大会报告工作，请予审议，并请市政协委员和其他列席会议的同志提出意见。

一、改革创新、稳步前进的2014年

刚刚过去的2014年是不平凡的一年。我们在中共烟台市委的正确领导下，认真贯彻落实党中央国务院、省委省政府一系列决策部署，扎实开展党的群众路线教育实践活动，全面深化改革，全面推进依法治市，积极作为稳增长、调结构、促改革、惠民生，经济社会发展取得了丰硕成果。

经济实现健康稳定增长。全市生产总值达到6002亿元，同比增长9.1%；城市和农村居民人均可支配收入分别达到35791元和16656元，分别增长8.6%和11.4%；城镇登记失业率3.22%；居民消费价格上涨1.9%。主要经济指标增幅高于全国、全省和14个首批沿海开放城市平均水平。经济增长质量和效益不断提高，产业层次逐步提升，区域结构进一步优化，财政收支结构得到改善。服务业占比提高1.4个百分点；一般公共预算收入490.2亿元，增长12.1%，税收占比提高3个百分点达到80.2%；连续9年完成节能任务。经济增长的内涵、动力和格局正发生新的变化。

重点领域工作取得新进展。农业领域，小麦、玉米新品种均创全国单产新纪录，粮食生产再获丰收，“海上粮仓”建设取得新成效，苹果邮票在我市首发。工业领域，10家企业进入全省纳税百强。通用东岳三款新车型投产，累计整车产量突破300万辆，动力总成产品突破900万台。万华工业园一期投产，成为全球最大的MDI供应商。中集来福士半潜式钻井平台占国内市场的80%、国际市场的近30%。杰瑞集团跻身全球石油天然气装备制造前三强。服务业领域，华海保险公司、海洋产权交易中心获批，绿叶制药、龙大肉食、杰瑞能服在境内外证券市场上市，9家企业在全国中小企业股份转让系统挂牌，29家企业在区域股权交易市场挂牌。我国首艘全资自主经营管理的邮轮“中华泰山”号成功运营。开通至韩国平泽客滚航线，港口货物吞吐量突破3亿吨，烟台港跃升全国化肥出口第一港。改革方面，成功获批并扎实开展创建国家电子商务示范城市、跨境贸易电子商务服务试点城市、国家海洋高技术产业基地试点城市等25个国家级、22个省级改革试点。开放方面，乐金显示三期投产成为全球最大手机显示屏生产基地，华恒租赁公司为全省最大外资融资租赁项目，36家企业首次实现境外投资，匈牙利宝思德经贸合作区成为首批省级境外经贸合作区。基础设施方面，全市人民翘首以盼的青烟威荣城际铁路济南方向通车，烟台蓬莱国际机场试飞成功，龙烟铁路加快建设，潍莱高铁、青岛至海阳城际铁路、环渤海高铁分别纳入近期及远期规划，烟台港西港区30万吨级矿石码头投入使用，至淄博液体化工输送管道基本完工，南北长山大桥顺利竣工，长岛实现从蓬莱跨海供水。城建方面，“规划一张图”管理系统获中国地理信息优秀工程金奖，建立“三房合一、租补分离”住房保障机制，创新城市管理模式。社会民生方面，市县两级承诺的为民服务实事全面完成，“四德”工程、志愿服务享誉全国，入选全国水生态文明城市建设试点市、中国最具生态竞争力城市。县域经济方面，龙口、莱州入选全省县域经济科学发展试点，福山、牟平和龙口、莱州、招远、蓬莱分别进入全国综合实力百强市辖区和县市。各个领域各条战线亮点纷呈，改革发展的动力在酝酿、能量在积蓄、优势在强化。

一年来，我们主要抓了以下几方面工作：

（一）多策并举稳增长，培育有利于相互对冲和平衡的新的经济增长支撑点。千方百计激发市场主体活力，改革工商注册登记制度，集中推出一批促进创业发展的政策措施，新登记市场主体8.9万户，增长93.7%，电子商务产业园、大学生创业园、金融超市等特色园区培育引进了大量代表新模式、新业态的成长型企业。努力增强投资拉动力，加快推进200个市级重点项目，张裕工业园顺利建设，海阳核电、南水北调配套工程如期推进，中粮大悦城、万达广场等一批服务业项目建成运营，固定资产投资4101亿元，增长15.9%。深入挖掘消费潜力，新建了一批肉食、蔬菜、早餐网点和商贸综合体，改造了一批农贸市场，启动建设肉菜流通追溯体系。积极引导信息交易和消费，全市85%的规模以上企业开展电商业务，电子商务交易额1565.6亿元，增长37.4%。社会消费品零售总额2377.7亿元，增长12.7%。实施外贸稳增长调结构措施，实现进出口527.5亿美元，增长7.6%，对全省外贸增长贡献率达到35.4%。成功承办省工商联商会建设经验交流会暨助推烟台海洋经济发展大会，积极参与全国工商联十一届三次执委会暨全国知名民营企业入鲁助推转调创洽谈会，广泛开展招商引资“大走访”活动，促成224个内资项目签约、422个外资项目增资或新设，全市实际使用外资17.7亿美元，增长10.2%。参加中韩经贸合作高层论坛，协办第20次中韩经贸联委会和中韩FTA扩大经济合作研讨会，与韩国群山市建立经济合作伙伴关系。着力增强区域发展带动力，深入实施山东半岛蓝色经济区、黄河三角洲高效生态经济区建设战略。海洋产业产值增长20%。东部新区海洋经济发展中心基本完工，110多个基础设施项目快速推进，海德新能源汽车产业园等10个过亿元产业项目开工建设。莱州湾建设过亿元项目71个。丁字湾项目总投资过千亿元。龙口湾人工岛群主体完工。长岛人均旅游收入居全国12个海岛县首位。烟台开发区综合实力保持国家级开发区第6位，高新区获批全国首批创新人才培养示范基地、中德低碳生态试点示范城市，保税港区成功复制上海自贸区部分创新制度，招远开发区加快转型升级步伐，昆嵛山自然保护区生态保护取得新进展。

（二）精耕细作调结构，构建以质量和效益为中心的现代产业体系。深入推进“抓产业促发展”，制定实施32个产业行业发展规划和扶持意见，促进29个行业转型升级，一些传统产业看到了新的发展前景。农业创新步伐加快，肉蛋奶产量保持稳定，新增高效农业节水灌溉面积20万亩，规模以上农业龙头企业销售收入过千亿元。龙口粉丝评为国家非物质文化遗产，新增5件中国驰名商标、5件国家地理标志证明商标。新增10个山东名牌、9个山东服务名牌、1个省长质量奖。工业实现提质发展，36个工业大类中有33个实现增加值增长，五大传统优势产业、五大战略性新兴产业、十大产品集群总体实力进一步增强。重点调度的100个过亿元技改项目加快实施，现代汽车、富士康研发中心等一批研发平台顺利建设。新增省级示范工程技术研究中心20家、企业院士工作站6家、企业技术中心17家，5个项目获得国家科学技术奖励，4项专利获中国专利奖。引进各类人才3.2万人。中科院沈阳计算技术研究所烟台分所落户。规模以上工业企业主营业务收入1.5万亿元，增长7.1%，高新技术产业产值占比达到40.7%。服务业扩容升级并进，金融业在综合措施推动下快速发展，4家银行新设烟台分行，新增村镇银行2家、新型金融组织42家、民间资本管理公司35家，新设立文化投资基金等一批产业基金。确立打造养老服务业典范城市和医疗健康产业基地式城市的发展定位，引发市场积极反应，福山乐天养老中心、瑞康健康服务产业园等20多个大型民营综合体落地建设，229处公立和民营养老机构床位达到4.6万张，成为全国健康养老服务业试点市。新增3个4A级景区，新投入运营海立方欢乐水世界等14个重点旅游项目，蝉联全国避暑旅游城市首位，获批赴台个人游试点城市，接待游客5481.5万人次，增长9.6%。航空旅客吞吐量431万人次，增长18.4%。获得中国鲁菜之都和全国优秀会展城市称号。

（三）积极稳妥促改革，着力激发体制机制活力。认真执行国家、省改革政策，扎实推进我市各领域改革。实施市县政府机构改革，市级行政审批事项精简49.8%、行政权力事项压减48.7%，公布了市级政府部门权力清单。深化财税体制改革，全面落实“营改增”等政策，共为企业减负15.7亿元。市县两级政府预决算、部门预决算、“三公”经费预决算首次公开。推进国有企业改革，市管企业通过商业模式创新形成了新特色新亮点。整合新农合和城镇居民医疗保险，6个县市公立医院综合改革试点扎实推进，新增248个村卫生室实施基本药物制度。土地承包经营权确权登记颁证工作顺利开展，市级和3个县市农村产权交易中心挂牌运营。启动4家农村信用社银行化改革，蓬莱、栖霞改革方案获银监会批复。供销社综合改革有序展开。事业单位绩效考核全面推进。我市被列为全国教育装备综合改革试验区。

（四）加大投入惠民生，努力让群众分享改革发展成果。城镇化质量指数居全省前列。实施714项城建工程，96条道路竣工，新建改建农村公路1412公里，8项供排水、垃圾处理工程完工。15个镇入选全国重点镇，25个村被评为国家、省传统村落或历史文化名村，96个村居被评为省级生态文明示范村，城乡环卫一体化基本实现全覆盖，农村生产生活环境明显改善。开展大气污染整治，实施减排工程294项，淘汰黄标车6.1万辆，“蓝天白云”天数继续保持全省第二。实施饮用水源地保护规划、河道环境综合治理，26条重点河流44个监测断面水质达标率稳定在85%以上。启动国家森林城市创建工作。创立“89000”民生服务平台，各县市区基本建成投入使用。发放小额担保贷款和创业补贴4.9亿元。新增城镇就业18.8万人，农村劳动力转移就业3.8万人。企业退休人员养老金、居民基础养老金、失业保险金、城乡低保、农村五保供养标准进一步提高。建成保障房6222套、新筹建2435套，改造棚户区2.36万户。实现农村贫困人口脱贫3.1万人，救助困难群众6.3万人次，为困难家庭学生发放资助款近2亿元。完成192项库区惠民工程。两所中等师范学校合并建成烟台高级师范学校并成立鲁东大学教师教育学院，烟台二中高中部顺利迁建并实现六区招生，133所乡镇公办中心幼儿园投入使用。毓璜顶医院成为山东省区域医疗中心，滨州医学院烟台附属医院建成投用。胶东红色文化建设成效明显,大型纪录片《胶东烽火》在中央电视台播出，电视剧《大秧歌》开拍，胶东革命烈士陵园、地雷战纪念馆入选国家级抗战纪念设施和遗址名录。市及莱州、长岛、龙口、牟平、蓬莱、招远再次荣获全国文化先进单位。率先完成体育设施“村村有”工程，烟台籍运动员在仁川亚运会上获得5金5银2铜好成绩。村（居）委会换届选举工作有序推进。健全和落实文明城市创建长效机制，扎实推进乡村文明行动，城乡文明程度进一步提高。12人获评全国孝亲敬老之星。再次荣获全国公益慈善百强城市称号。人口计生工作在全省考核中实现七连冠。信访、综治、维稳工作扎实有效，社会治安、食品药品安全形势总体稳定，安全生产事故起数、死亡人数连续13年“双下降”。对口支援新疆、西藏等工作取得新成效。国防动员、民兵预备役、人民防空和双拥共建工作顺利开展，妇女、儿童、残疾人、红十字事业健康发展，统计、史志、档案、仲裁、机关事务、新闻出版、广播电视、民族宗教、气象、地震等工作取得新成绩。

（五）持之以恒转作风，进一步加强政府自身建设。群众路线教育实践活动使政府工作人员经受了深刻的思想洗礼，促进了党风政风的明显好转，形成了推动改革发展的强大动力。严格落实中央八项规定精神和省委实施办法、市委实施意见，集中解决“四风”问题，全面办理从各个方面征集到的意见建议，修订和新建了政府法律顾问制度等一系列规章规则。坚持把政府工作置于人大及其常委会监督之下，严格执行人大及其常委会的决议、决定，及时报告重要事项，自觉接受专题询问。主动向政协通报情况，重大决策实行民主协商。积极提请并全力配合人大、政协专题视察，办复市人大代表建议154件、政协提案516件。加强行政监察和审计监督，廉政建设和反腐败工作得到加强。

各位代表，过去一年取得的成绩实属不易。这是党中央国务院、省委省政府和市委科学决策、正确领导的结果，是市人大、市政协和社会各界有效监督、支持参与的结果，是广大干部职工和全市人民真抓实干、共同努力的结果。在此，我代表市人民政府，向全市人民，向市人大代表、政协委员，向中央省属驻烟单位、驻烟人民解放军和武警部队官兵，向各民主党派、工商联、各人民团体、社会各界人士和离退休老同志，向所有关心支持烟台发展的海内外朋友们，表示衷心的感谢和崇高的敬意！

各位代表，我市经济社会发展取得了一些成绩，但仍然存在不少困难和问题。经济下行压力较大，企业生产经营困难不少，后续项目储备不足，出口波动加剧，经济增速换挡回落；结构性问题仍然比较突出，产业升级任务很重，新兴产业尚未形成有力支撑；资源环境约束趋紧，节能减排任务更加繁重；安全生产和食品药品监管压力较大；公共服务离群众期望还有一定差距；有的政府机关和工作人员法治意识、服务意识仍比较薄弱，作风建设成果还不牢固，反腐倡廉任务依然艰巨。我们将勇于正视、积极应对这些问题。

二、2015年政府工作总体思路和目标任务

今年是全面深化改革的关键之年，是全面推进依法治国的开局之年，也是全面完成“十二五”规划的收官之年。政府工作的总体思路是：全面贯彻党的十八大和十八届三中、四中全会精神，以邓小平理论、“三个代表”重要思想、科学发展观为指导，认真贯彻落实习近平总书记系列重要讲话特别是视察山东重要讲话和重要批示精神，坚持稳中求进工作总基调，坚持以提高经济质量和效益为中心，牢牢把握“一个定位、三个提升”的要求，主动适应经济发展新常态，着力以改革开放的办法激活力、稳增长，着力以调整结构的举措补短板、提质量，着力以改善民生的成效增福祉、促和谐，努力保持经济社会持续健康发展。

经济社会发展的主要预期目标是：全市生产总值增长8.5%左右，一般公共预算收入增长10%左右，固定资产投资增长15%左右，社会消费品零售总额增长12.5%左右，进出口增长6%左右，实际使用外资平稳增长，城市和农村居民人均可支配收入分别增长9%和10%左右，城镇登记失业率控制在3.5%以内，居民消费价格涨幅控制在3%以内，人口自然增长率控制在2.2‰以内，完成节能减排任务，全面落实国家、省、市各类民生政策，保持社会和谐稳定，大力建设宜居宜商宜游的城乡环境，努力实现“十二五”经济社会发展各项目标。

各位代表，以上目标综合考虑了国内外经济形势，兼顾了需要和可能，体现了年度目标和五年规划目标相衔接。对此，我们有压力也有信心，一定要加倍努力，千方百计实现今年目标。

实现今年目标，必须深刻理解当前和今后一个时期我国经济发展的大逻辑，把握正确的方向。我们要把思想统一到中央对经济发展新常态的重大战略判断上来，认识新常态，适应新常态，引领新常态，努力做到调速不减势、量增质更优。我们要以习近平总书记对山东提出的“走在前列”的要求为目标定位，提升发展标杆，提升工作标准，提升精神境界，着力在深化改革扩大开放、转方式调结构、创新驱动发展、加强社会治理创新、弘扬优秀传统文化方面下功夫，力争更多工作走在全省全国前列。

实现今年目标，必须保持稳增长和调结构平衡，始终坚持以提高质量效益为中心。我们要促进消费、投资、出口“三驾马车”更加均衡地拉动经济增长，充分发挥消费的基础作用，打造多点支撑的消费增长格局；充分发挥投资的关键作用，着力推进内涵效益型投资增长；充分发挥出口的支撑作用，加快形成以技术、品牌、质量、服务为核心的外贸竞争新优势。我们要按照“向蓝、登高、望远”的思路，打好转方式调结构攻坚战，促进信息化与工业化、生产性服务业与一二三产业深度融合，提高现代服务业和战略性新兴产业比重，加快推进农业现代化。我们要大力实施区域发展战略，积极融入国家“一带一路”战略，围绕建设“蓝黄”两区，大力推动“一极领先、多极崛起”板块突破发展，乘势而上培育铁路经济增长带、建设空港经济区、发展海港产业园，促进东部新区尽快发展成转型升级高地，在更高水平上建设烟台开发区和招远开发区的开放高地、高新区的创新高地、保税港区的政策高地、昆嵛山自然保护区的生态高地，继续帮扶栖霞、支持长岛科学发展，推动各个区域激活力、出亮点、求突破、见成效。

实现今年目标，必须推进改革创新，加快依法治市进程。我们要加强对经济体制改革的深度谋划和系统推进，突出重要领域和关键环节，集中突破重点、难点，挖掘改革产生的新动力、新红利。我们要用好两种资源、两个市场、两类规则，既重视对国外的开放，又加强对国内市外的合作。我们要加快从要素驱动、投资驱动发展转变为创新驱动发展，全面推进科技创新和商业模式创新。我们要创新社会治理机制，实现政府治理和社会自我调节、居民自治良性互动。我们要运用法治思维和法治方式开展工作，在创造经济繁荣的同时创造法治文明。

实现今年目标，必须动员方方面面的力量，形成强大工作合力。每一个烟台人都是烟台发展的见证者、参与者、受益者，都是新一轮改革发展的拥护者、实践者、推动者。我们要在市委的坚强领导下、在市人大和市政协的监督支持下更加努力地工作，与广大企业一道共同奋斗，与全市人民一起建设幸福家园，众志成城开创烟台美好的明天。

各位代表，当前经济增长正在发生具有中长期意义的重要变化，今年改革发展的道路不会一帆风顺，困难可能会有不少。我们一定要强化战略思维，保持冷静心态；坚持底线思维，做到理性研判；增强机遇意识，激发进取精神，努力在新常态下继续拿到好成绩，实现“十二五”圆满收官，满怀信心地跨入“十三五”。

三、在新常态下保持经济平稳健康发展

针对我市经济发展的形势和阶段性特点，集中精力突破主要矛盾和矛盾的主要方面，积极发现培育新的增长点，在稳增长中不断把转方式调结构引向深入。

（一）深入推进“抓主体促发展”，动员方方面面支持和参与创业创新创富。围绕激发市场主体活力综合施策，为就业者、创业者、投资者提供最便利的服务和最有效的支持。通过激发创业热情新生一批本土市场主体，扶持新拿到入场券的小微企业和个体工商户站稳脚跟、发展起来，让更多的人看到希望、踊跃跟进；通过积极招商引进一批外来市场主体，使更多的国内外优秀企业到我市创业发展；通过推动主辅分离再生一批市场主体，支持农业和制造业骨干企业剥离配套服务业，培育专业化、社会化、规模化的服务业市场主体；通过促强扶弱壮大一批市场主体，继续支持“个转企”、“小升规”、“规改股”、“股上市”，鼓励龙头企业、上市公司建设专业化园区；通过支持市场化并购重组整合一批市场主体，促进企业扩能升级或浴火重生；通过引导企业“走出去”强化研发和开拓市场发展一批市场主体，培育更多源自烟台的全国性企业和跨国公司。

（二）深入推进“抓产业促发展”，把转方式调结构工作做细做实。按照“敲开核桃”作分析和“一行业一对策”找思路的要求，促进传统行业转型升级、潜力行业破茧成蝶、嫩芽行业长大成材，尤其是大力推动系统重要性行业加快发展。农业转型升级的方向是加快从注重生产向生产、经营并重转变。推动粮油、水果、水产、畜牧、蔬菜等主导产业通过保安全、提质量实现生产升级，培育茶叶、休闲观光农业等特色产业，推进“海上粮仓”建设，做大做强种子种苗产业，放大烟台农产品、水产品和食品的品牌优势，把产业链、价值链等现代产业组织方式引入农业。加快旱涝保收高标准农田建设。提升农业机械化水平。抓好动物无规定疫病区、肉菜流通追溯体系建设，巩固出口农产品质量安全示范市建设成果。加快培养新型职业农民队伍，培育发展农民合作社、家庭农场、专业大户等新型农业经营主体，促进农业龙头企业集群式发展。探索建设特色优势农产品以及农业生产资料的生产、集散、交易、信息、价格和研发中心，打造农资、农技、农副、金融“四位一体”的服务平台。工业转型升级的方向是由整体转型向各子行业升级发展。继续实施“5510”和“8515”工程，促进计算机和通信以及其他电子设备制造业等5个主营业务收入千亿元行业由强向精发展，促进通用设备制造业等18个过百亿元行业由大向强升级，促进化学纤维制造业等13个低于百亿元行业由小向大迈进，加快建设汽车及零部件、聚氨酯、创新药物研发和产业化等15个乃至更多的产业基地，重点打造食品名城、国际葡萄·葡萄酒城、黄金产业名城、节能环保产业城等4个乃至更多的产业名城。积极培育战略性新兴产业，推进海工装备、生物科技、航空材料、核电装备制造等高端产业园区建设，重点突破智能装备、卫星应用、物联网应用、云计算、新型显示等关键领域，培育发展个性化定制、众包设计等新业态，聚集发展应急管理、公共安全等特色产业。抓好上汽变速器总成等100多个过亿元技改项目，推动市场空间大的工业企业扩大产能、升级换代。服务业转型升级的方向是推动生产性服务业向规模化发展、生活性服务业向便利化发展、公共服务业向均等化发展。顺应制造业服务化新趋势，重点培育发展金融保险、现代物流、电子商务、研发设计、法律会计等生产性服务业。顺应居民消费新特点，抓好生活性服务业布局。抓住交通条件改善的重大机遇加大旅游营销力度，加快推进重点旅游项目，建设机场、车站旅游咨询与集散中心，大力发展滨海旅游、乡村旅游，打造优秀休闲度假城市。完善商贸流通体系，促进住宿餐饮业转型发展。推动房地产业平稳健康发展。顺应公共服务新需求，提升教育、文化、体育、医疗、养老等公共服务业发展水平。大力推进标准化建设，支持企业参与制定国家标准和行业标准，努力提升产品质量、工程质量、服务质量，创造更多烟台优势品牌。

（三）深入推进“抓改革促发展”，创造动力并收获新一轮改革发展红利。积极借鉴上海自贸区改革试点经验，在保税港区复制海关监管和检验检疫制度等创新措施，在高新区争取实施中关村试点政策。深化国有企业改革，推进产权多元化，发展混合所有制经济，逐步建立以管资本为主的国有资产监管模式。深化资源性产品价格改革，建立健全居民用水、用气阶梯价格制度，构建烟台特色优势产品价格指数体系。深化财税体制改革，今年努力使预算管理、国库管理、政府债务管理改革取得决定性成果，财政支出管理改革取得实质性突破，税制改革取得阶段性成效。深化投融资体制改革，积极推广投资基金、政府购买服务、政府与社会资本合作等多种投融资方式，逐步建立基础设施建设运营的市场化机制。深化农村综合改革，基本完成土地承包经营权确权登记颁证工作，探索土地承包经营权流转、抵押、担保等办法，稳妥开展新型农村合作金融试点，扩大政策性农业保险覆盖面，实施海域使用权市场化配置。继续推进供销社综合改革试点。全面启动教育综合改革，积极稳妥实施校长职级制改革和招生制度改革。深化医药卫生体制改革，扩大公立医院改革范围，巩固完善基本药物制度和基层运行新机制，促进基本公共卫生服务均等化。深化户籍管理制度改革，逐步解决农业转移人口和外来务工经商人员市民化问题。

（四）深入推进“抓开放促发展”，推动开放向优化结构、拓展深度、提高效益的方向转变。全面扩大对内对外开放，深化面向市外国内的开放，纵深推进产业国际化、市场国际化、企业国际化、园区国际化、城市国际化，高水平引进来、有秩序走出去。坚持“大走访”常态化，深化与央企、省企、外企、民企、大学和科研院所的个性化、“量身定做”式合作，强化与国际一流投融资机构、咨询机构、会计师事务所、律师事务所等在委托招商、中介招商方面的长期合作，鼓励境外上市、境外发债等多种引资方式。扎实开展主题招商，既注重引进项目，又注重引进技术和人才。积极扩大农业、科技、教育、卫生、文化、体育、养老等方面对外开放。促进外贸转型升级，加大对骨干外贸企业扶持力度，鼓励中小微型企业从事对外贸易，支持创建境外市场公共销售平台，鼓励融资租赁、跨境电子商务、工程带动等新兴出口方式，培育市场采购贸易、外贸综合服务等新平台，发展服务贸易、服务外包等新业态，促进高新技术和机电产品出口扩规模上层次。建设进口商品展示交易中心和专营网络，加大品牌引进力度，支持能源资源性产品、先进技术设备、关键零部件和与群众生活密切相关的一般消费品进口。完善电子口岸，提高贸易便利化水平。支持有条件的企业构建全球化生产营销体系，建立海外研发机构和资源供应基地。加快外事工作转型发展。拓展与韩日两国及东南亚、港台两地、欧美两洲、京沪深、环渤海区域的合作，努力打造中韩地方经济合作的先行区和示范区，积极探讨中新合作开发龙口裕龙岛石化产业园区，深入谋划建设“一带一路”战略支点城市的工作措施。筹备召开第五届东亚经济交流推进机构总会，办好烟台国际葡萄酒博览会和国际果蔬·食品博览会。推动各类园区在继续扩大经济总量的过程中努力实现由追求速度向追求质量转变，由政府主导向市场主导转变，由同质化竞争向差异化发展转变，由硬环境见长向软环境取胜转变。

（五）深入推进“抓创新促发展”，让更多的人、更多的企业展现创新活力。大力推动以企业为主体、以技术创新和产品创新为核心的科技创新。积极参与山东半岛国家自主创新示范区建设，完善支持企业创新的财政、金融和政府采购等政策，调整科技计划支持导向，鼓励应用性技术研发、战略性新兴产业发展、创新平台建设。设立天使基金、成果转化引导基金，吸引社会资本参与科技创新及科技成果产业化。不断深化产学研协同创新机制，推动企业与大学和科研院所组建产业技术创新联盟。加强产业导航服务平台、大型科学仪器设备共享平台建设，科技企业孵化器达到50万平方米以上。设立专利奖，提升国家知识产权示范城市建设水平。围绕创新设计和制定持久性人才政策，完善人才培养、引进、使用和激励机制，引导人才向产业和科研一线流动。鼓励企业开展商业模式创新，积极发展平台经济，规范整合化工产品、葡萄酒、苹果、海产品等交易品种，探索建立介于现货与期货之间的综合性大宗商品交易平台，推动海洋产权交易中心早日运营，促进联合产权交易中心做大规模。加快电子商务示范城市、跨境贸易电子商务服务试点城市建设，培强做大电商载体。引导工业企业由生产制造商向以制造为基础的综合服务商转型，推动服务业企业积极拓展新业态新模式。鼓励产业互联和有序的跨界融合，积极推动产业、金融、科技深度融合发展。在防范风险的基础上大力推动金融创新，让企业和居民个人享受更多更好的金融服务。建设区域性私募市场和基金管理中心，压减财政一般竞争性领域专项资金，设立政府引导基金，引入社会资本建立一批产业投资基金，尽快形成规模较大、实力较强的基金群。加快恒丰银行、烟台银行综合改革，推进县级农村信用社改制为农村商业银行，促进民间融资机构和村镇银行、小额贷款、融资担保等普惠金融组织健康发展。推动企业境内外上市，鼓励更多企业在场外市场挂牌。强化金融监管和金融生态建设，打击非法集资，主动防控和有效化解金融风险。

（六）深入推进“抓环境促发展”，建设一流的营商环境。围绕打造软硬环境、促进投资便利化，着力在搞好信息服务、改善营商环境、完善基础设施、增强配套能力、维护公平竞争上下功夫。落实好减轻小微企业和高新技术企业负担的减税、降费政策。统筹协调煤电油气运等生产要素，保障企业正常生产经营。尊重企业，尊重企业家，尊重产业工人，充分发挥各类专业技术人员和职业经理人作用。引导行业协会、商会等市场中介组织做好服务和自律工作。扎实解决制约行业发展的共性问题和关键问题，分类解决制约企业发展的人才、技术创新、土地、资金以及市场推广等具体问题。完善企业化职业教育模式，多层次培养工程师、技师等高素质劳动者。优化各级政务服务机构运行，规范完善公共资源交易平台。加快建设智慧城市，促进数据互联互通共享。健全社会信用体系，探索建立公共信用信息交换和共享平台。强化对市场全过程监管，营造公平有序的市场竞争环境，真正让市场既有活力、有动力，又有秩序、有责任。

各位代表，抓主体促发展、抓产业促发展、抓改革促发展、抓开放促发展、抓创新促发展、抓环境促发展是针对当前我市经济发展特点探索形成的工作措施，我们将根据形势变化，不断充实强化“六抓”促发展具体事项，更好地促进我市经济行稳致远。

四、推动民生与社会事业全面发展

经济发展了，各个方面和每个人就有更多的空间去分享，我们要建立科学合理的资源和财富分配机制，让发展成果更多更公平地惠及全市人民。

（一）持续增加城乡居民收入。深化收入分配制度改革，健全资本、知识、技术、管理等由要素市场决定的报酬机制。保障劳动者权益，加强对企业工资分配指导，推进同工同酬，支持完善激励约束机制。按照上级统一部署，改革机关事业单位工资制度，调整工资标准，优化薪酬结构，在县以下机关全面实行职务职级并行制度，进一步提高基层公务员工作补贴，实施养老保险制度改革。继续落实大学生就业促进计划和创业引领计划，开展农民工职业技能培训提升、权益保障和公共服务三年行动，开发公益性岗位4000个以上，帮扶5000名以上城镇困难人员实现再就业，新增城镇就业13万人以上，农村劳动力转移就业2.5万人以上。搭建创业平台，发挥小额担保贷款和创业补贴作用，以创业带动就业。加大对农业、农村、农民的支持力度，引导城市现代生产要素向农业农村流动，推动公共服务向农村延伸，做好精准扶贫工作，保持农民收入稳定增长。

（二）持续完善公共服务保障体系。深入实施素质教育，让孩子们都能享受优质均衡的义务教育，让贫困家庭的孩子都能接受公平的有质量的教育。推进175所义务教育学校标准化建设。加快发展学前教育，推动高中教育特色发展，构建现代职业教育体系，支持在烟高校发展，提升特殊教育水平，鼓励发展民办教育、国际教育。优化医疗资源布局，注重医疗业态升级，完善卫生服务体系，加快多元化办医进程，打造5公里预防接种服务圈。实施全民健身计划，增加群众活动场所，提高竞技体育水平。深入推进“89000”民生服务平台建设。按照国家和省的部署，不断提高退休人员基本养老金、居民基础养老金、居民医保财政补助、城乡低保、农村五保供养标准，职工医疗保险大额救助金最高支付限额由15万元提高到40万元，将生育引起疾病和合并疾病纳入生育保险保障范围，进一步健全社会救助体系。探索建立保障性房源多元化筹建方式，新筹建保障性住房800套，改造一批棚户区。大力发展医养结合型养老机构，新增养老床位3000张以上，打造15分钟社区养老服务圈。完成200项库区惠民工程。搞好人口计生服务，保障老年人、妇女、儿童和残疾人权益，扎实推进史志档案、民族宗教、仲裁、红十字、慈善等各项工作。开展人民防空进社区工作，推动军民融合深度发展，加强国防教育、国防动员、民兵预备役和双拥共建、优抚安置工作，积极创建双拥模范城。

（三）持续改善城乡面貌。坚持走具有胶东特色的功能区带动型城镇化道路，建设都市化的中心城市、特色化的县域城市、风情化的小城镇和田园化的美丽乡村。推动中心城市扩容升级，加快专项规划和控制性详细规划修编，推进重点区片开发，实施城中（郊）村改造，续建、新建263项城建重点工程。提升县域城市发展水平，建设富有特色、充满活力、宜居宜业的小城市。深入开展“扩权强镇”试点，培育一批旅游型、工业型、生态型、商贸型特色小城镇。扎实开展生态文明乡村和农村新型社区建设，保护历史文化名村和传统村落，深入推进农村环境整治。加强城乡基础设施建设，实现烟台蓬莱国际机场转场运营，加快龙烟铁路建设，推进蓬栖、龙青、文莱高速公路和原油码头、电厂、天然气等项目，促进海阳核电二期工程核准开工，开展潍莱高铁、城市轨道交通前期工作，大力发展公共交通。实施全面普查，建立地下管线综合管理信息系统。推进病险水库除险加固工程，完成南水北调配套工程主体建设任务。新增天然气用户6万户、供热面积220万平方米，改善15万农村人口饮水条件。我们将重点建设惠及全市人民的基础设施，全面提升精细化管理水平，让人们出行时更多地感受到基础设施的通达，让人们打开水龙头时更多地感受到基础设施的细润，让人们在寒冷的冬夜更多地感受到基础设施的温暖。

（四）持续优化生态环境。高度关注空气质量，继续强化大气污染防治措施，开展建筑工地施工扬尘、城市裸露渣土和交通扬尘、燃煤污染、机动车排放污染、油气污染等5个专项治理行动，完成6.8万辆黄标车淘汰任务，取缔市区禁燃区120台高污染燃料燃用设施，努力争取更多的“蓝天白云”天数。高度关注水质状况，加强水源地环境保护，落实10大类23项重点整治工程，全面治理重点河流。加大节能减排力度，推行工业节能、绿色建筑、绿色交通等计划，推广循环经济和清洁生产先进技术，大力发展节能环保产业，完善能源管理体系和能耗量交易机制。保卫和创造绿色财富，以创建国家森林城市、全国水生态文明城市、全国海洋生态文明示范区为抓手，开展城郊森林建设等造林工程，加强水系生态建设，推进海洋生态修复治理，落实最严格的耕地林地湿地保护和水资源管理制度。强化生态倒逼机制，完善主体功能区制度，实现经济社会与生态环境的双赢发展。

（五）持续增强文化软实力。围绕构建社会主义核心价值体系，抓住纪念抗战胜利70周年的机遇，加大对胶东文化特别是胶东红色文化的挖掘、阐发和宣传力度，建设胶东红色文化龙头城市。讲好烟台故事，叫响国家历史文化名城品牌。积极创建国家公共文化服务体系示范区，培训基层群众文艺骨干，广泛开展文化惠民活动。大力推进文明城市建设，深入实施“四德”工程，以文明的力量提升城市竞争力。把文化事业与文化产业发展紧密结合起来，促进文化创意等新兴业态与旅游、体育、养生、科技等产业深度融合，为广大群众提供更加优质的文化产品和服务。

（六）持续巩固和谐稳定局面。深入开展法治烟台、平安烟台、和谐烟台“三城联创”活动。围绕建设法治烟台，健全公共法律服务体系，以“法德共进”活动为创新载体深入开展法治宣传教育，努力营造办事依法、遇事找法、解决问题用法、化解矛盾靠法的法治环境。围绕建设平安烟台，推动以案件事件为中心的安全观向以人为中心的安全观转变，深化社会治安综合治理，加强禁毒工作，实施“天网”工程，推行社区网格化管理。把安全生产摆到更加突出位置，健全“党政同责、一岗双责、齐抓共管”的责任体系，全面彻底排查整治安全隐患，深化“平安行·你我他”行动，坚决杜绝重特大安全事故发生。从源头上加强食品安全监管，开展“食安烟台”建设和国家食品安全城市创建试点，保障饮食用药安全。强化应急管理，抓好气象服务和防震、防汛、防台风及抗旱减灾、森林防火、海上搜救等工作。围绕建设和谐烟台，充分发挥工会、共青团、妇联等群团组织，以及各种专业协会和志愿者组织等在社会治理中的重要作用。以法治为引领深入推进信访工作制度改革，有效化解社会矛盾，打造祥和的氛围。

各位代表，持续增加城乡居民收入、持续完善公共服务保障体系、持续改善城乡面貌、持续优化生态环境、持续增强文化软实力、持续巩固和谐稳定局面，是促进经济社会协调发展的主要措施。为人民群众谋福利是我们一切工作的出发点和落脚点，尽管今年经济形势可能造成地方财政收入增速放缓，但是民生支出的规模一定会稳定提高，我们要花好用于民生保障的每一分钱，用改善民生的新成果兑现发展为民的理念和承诺。

五、全面加强政府自身建设

我们要进一步解放思想、锐意进取，积极开辟保持经济社会持续健康发展的新途径。实现“走在前列”的目标定位，既是我们的光荣使命，更意味着责任和担当，必须付出更多的艰辛、更多的汗水。我们一定要增强新常态下改革创新、干事创业的进取意识，鼓足凤凰涅槃、浴火重生的勇气，保持知难而进、奋发有为的精神状态，牢记使命，勇于担当，在更大的格局中来定位，精心编制“十三五”规划，以新视角把脉经济，以新思维拓展新局，以新境界新作为推动经济社会发展稳中有进、稳中向好。

我们要进一步转变职能、简政放权，努力创造审批环节少、办事效率高、行政成本低的政务环境。按照“清权、减权、优权、制权”的思路，承接上级下放权力，做好向市场放权、向基层放权、向社会移权工作。梳理行政审批事项目录清单、行政权力清单、政府责任清单、市场准入负面清单，完善政府服务平台，推行“一口受理”、实现“限时办理”、严格“规范办理”、坚持“透明办理”、推进“网上办理”，加强事中事后监管。实施事业单位分类改革，推进法人治理结构建设。

我们要进一步依法行政、科学决策，加快建设职能科学、权责法定、执法严明、公开公正、廉洁高效、守法诚信的法治政府。认真执行市人大及其常委会决议、决定，做好人大代表建议和政协提案办理工作，自觉接受人大、政协监督，主动接受司法监督、舆论监督和社会公众监督，强化监察、审计监督和政府内部层级监督。从今年开始向市人大报告全口径预算。严格执行《烟台市重大行政决策程序规定》、《烟台市人民政府议事规则》等规章制度，提高科学、民主、依法决策水平。全面推进政务公开，完善政府信息发布制度，及时回应社会关切。

我们要进一步改进作风、勤政务实，使作风建设落地生根成为常态。巩固和拓展群众路线教育实践活动成果，严格执行中央八项规定精神和省委实施办法、市委实施意见，持之以恒地反对“四风”，坚决治理“为官不为”。从严控制“三公”经费和一般性支出，推进公务用车改革。牢记全心全意为人民服务的宗旨，通过一件件小事的办理、一件件实事的落实、一件件难事的解决，赢得人民群众的信任和支持。

我们要进一步严明纪律、从严治政，保持惩治腐败高压态势。严格履行党风廉政建设责任制，坚持党风廉政建设与经济社会发展一起谋划、一起部署、一起考核，健全完善惩治和预防腐败体系，严肃查处违法违纪案件，树立为民务实清廉的良好形象。

各位代表，经过一代又一代烟台人的不懈努力，去年我市生产总值占到全国的近1%，9项主要经济指标总量在全国大中城市中居第14位到第25位之间，增速居第5位到第15位之间，一些工作走在全国前列。我们对烟台发展的成果感到自豪，对未来充满信心。当然，前进中的困难、风险和挑战不容忽视，我们必须积极应对。现在我们比任何时候都需要改革创新。习近平总书记强调，惟改革者进，惟创新者强，惟改革创新者胜。让我们紧密团结在以习近平同志为总书记的党中央周围，在中共烟台市委的坚强领导下，解放思想、开拓奋进，努力走在全省全国前列，为全面建成小康社会、率先基本实现现代化，早日把烟台建设成为蓝色文明幸福之都而努力奋斗！
